# Supplementary figures and images for: A synthetic method to assay polycystin channel biophysics
Source: eLife. 2024 Oct 28;13:RP98534. doi: 10.7554/eLife.98534 (PMC11517255; doi:10.7554/eLife.98534)

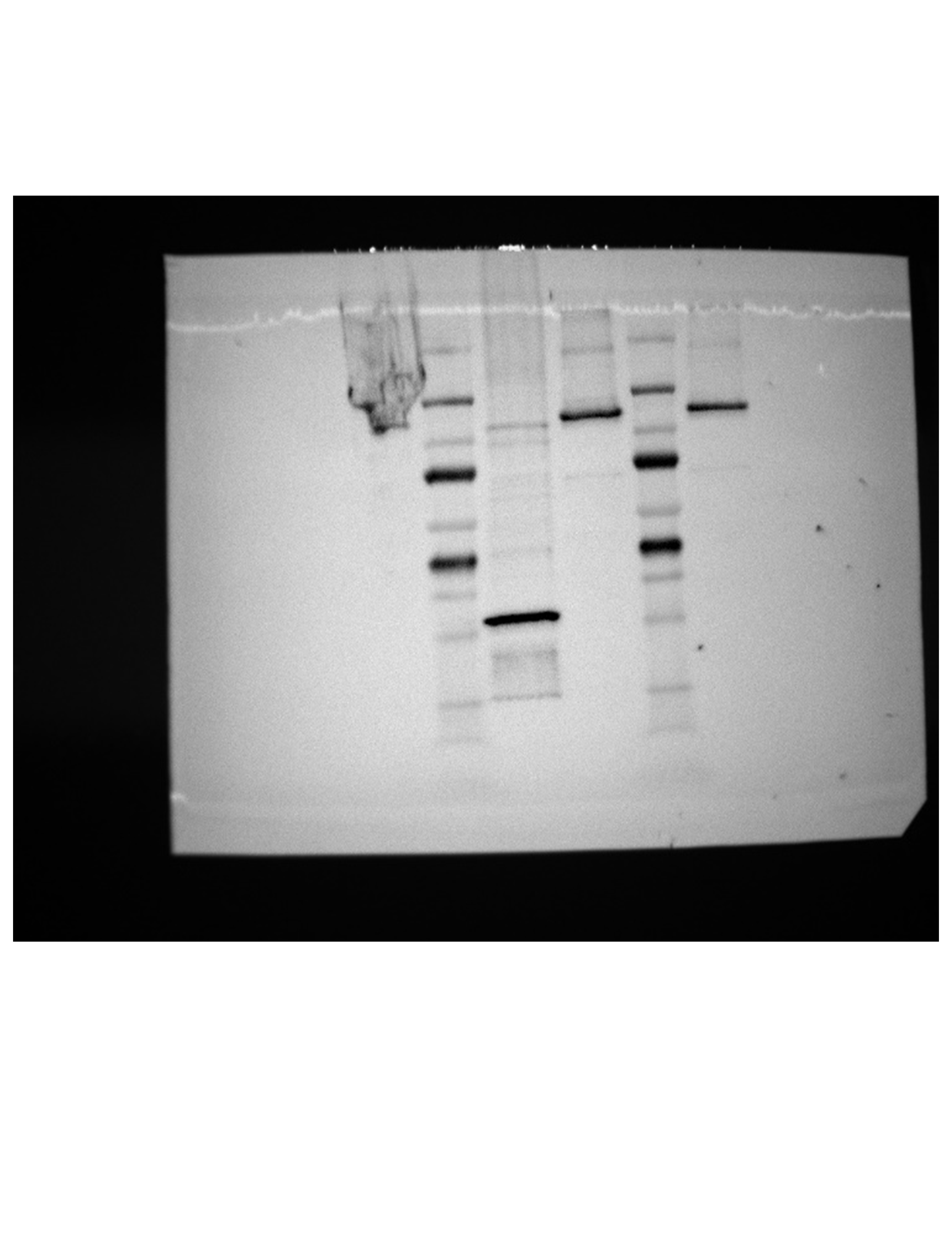

Supplement: Figure 1—source data 1. [file elife-98534-fig1-data1.zip › Figure_1_Source_Data_1.tif]
