# Supplementary material for: A synthetic method to assay polycystin channel biophysics
Source: eLife. 2024 Oct 28;13:RP98534. doi: 10.7554/eLife.98534 (PMC11517255; doi:10.7554/eLife.98534)
Supplement: Figure 1—source data 2. [file elife-98534-fig1-data2.zip › Figure_1_Source_Data_2.pdf]

Western blot

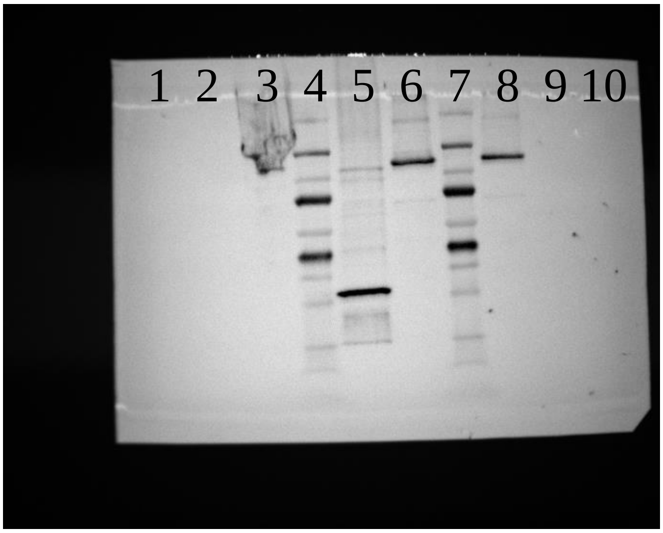

<u>Wells</u>

1. Empty
2. Empty
3. PKD2L1-GFP 15µl
4. SPECTRA protein ladder
5. GFP 3µl
6. PKD2L1-GFP 7µl
7. SPECTRA protein ladder
8. PKD2L1-GFP 5µl
9. Empty
10. Empty
